# Differential Immunometabolic Phenotype in Th1 and Th2 Dominant Mouse Strains in Response to High-Fat Feeding

**DOI:** 10.1371/journal.pone.0134089

**Published:** 2015-07-28

**Authors:** Nemanja Jovicic, Ilija Jeftic, Ivan Jovanovic, Gordana Radosavljevic, Nebojsa Arsenijevic, Miodrag L. Lukic, Nada Pejnovic

**Affiliations:** 1 Center for Molecular Medicine, Faculty of Medical Sciences, University of Kragujevac, Kragujevac, Serbia; 2 Institute of Histology, Faculty of Medical Sciences, University of Kragujevac, Kragujevac, Serbia; 3 Institute of Pathophysiology, Faculty of Medical Sciences, University of Kragujevac, Kragujevac, Serbia; College of Tropical Agriculture and Human Resources, University of Hawaii, UNITED STATES

## Abstract

Immune reactivity plays an important role in obesity-associated metabolic disorders. We investigated immunometabolic phenotype of C57Bl/6 and BALB/c mice, prototypical Th1 and Th2-type strains, fed chow or high-fat diet (HFD) for 24 weeks. In comparison to C57Bl/6 mice, chow-fed BALB/c mice had higher body weight and weight gain, lower glycemia, more pronounced liver steatosis, but less inflammation and collagen deposition in liver. In response to HFD C57Bl/6 mice exhibited higher weight gain, higher glycemia, HbA1c and liver glycogen content, increased amount of visceral adipose tissue (VAT) and number of VAT associated CD3^+^CXCR3^+^ T cells, CD11c^+^ dendritic cells (DCs) and F4/80^+^ macrophages than BALB/c mice. More numerous CD3^+^ and CD8^+^ T lymphocytes, myeloid DCs, proinflammatory macrophages (F4/80^+^CD11b^+^CD11^+^ and F4/80^+^IL-1β^+^) and CD11b^+^Ly6C^high^ monocytes and higher levels of proinflammatory IL-6, TNF-α and IFN-γ were present in liver in HFD-fed C57Bl/6 mice compared with diet-matched BALB/c mice. As opposed to C57Bl/6 mice, HFD induced marked liver steatosis and upregulated the hepatic LXRα and PPARγ genes in BALB/c mice. C57Bl/6 mice fed HFD developed liver fibrosis and increased hepatic procollagen and TGF-β mRNA expression, and IL-33, IL-13 and TGF-β levels in liver homogenates, while BALB/c mice fed HFD had scarce collagen deposition in liver. The obtained results suggest inherent immunometabolic differences in C57Bl/6 and BALB/c mice. Moreover, HFD Th1-type mice on high fat diet regimen are more susceptible to adiposity, liver inflammation and fibrosis, while Th2-type mice to liver steatosis, which is associated with differential immune cell composition in metabolic tissues. Strain-dependent differences in immunometabolic phenotype may be relevant for studies of obesity-associated metabolic diseases in humans.

## Introduction

Nonalcoholic fatty liver disease (NAFLD) is the leading cause of chronic liver diseases in developed countries and includes disorders characterized by increased lipid accumulation in hepatocytes which can range from benign liver steatosis to non-alcoholic steatohepatitis (NASH), liver cirrhosis and hepatocellular carcinoma [1]. Approximately in 20% of patients, NAFLD progresses to liver inflammation and its complications [2]. NASH is closely linked to metabolic syndrome and has more severe course in patients with type 2 diabetes [3]. Genetic and environmental factors play a role in the development of obesity, NASH and hepatic fibrosis [4–6], but the cellular and molecular mechanisms involved in the obesity-associated metabolic disorders are incompletely understood. Complex immune mechanisms and cytokines orchestrate inflammatory response and metabolic disturbances in this context [7–9]. The rodent model that fully reflects development of these metabolic diseases in humans is not defined [10].

A variety of mouse strains, knockout and transgenic mice, have been used in studies of obesity-related metabolic diseases. The reported genotype dependent differences in the development of fibrotic NASH [11] could be associated with differential immune and inflammatory responses to metabolic danger molecules. Accumulating evidence suggest that immune cell activation affects obesity-induced chronic inflammation, insulin resistance and diabetes [12].

C57Bl/6 and BALB/c mice are commonly used mouse strains for studies on immunoregulation in various disease models. As C57Bl/6 mice preferentially develop Th1 immune response and BALB/c mice Th2-type cytokine polarisation, they are regarded as protototypic Th1 and Th2-type mouse strains, respectively [13–16]. In addition to their distinct T-cell responses, macrophages from these two mouse strains exert different reactions in response to various stimuli [17]. Recent evidence indicates that the balance between M1/M2 macrophages and Th1/Th2 lymphocytes is of critical importance for the outcome of many diseases including obesity-related metabolic disorders [18]. In obesity, there is an increase in number of dendritic cells (DCs) and macrophages in adipose tissue and liver which contribute to metabolic alterations [19]. It is reasonable to speculate that the constitutive differences in the distribution of immune cells in metabolic tissues exist across mouse strains. However, the difference in immune responses in C57Bl/6 and BALB/c mice in relation to metabolic variables is not elucidated. These mouse strains are particularly attractive as several “knock out" mice for genes related to relevant molecules of innate and acquired immunity are available. Moreover, as demonstrated in recent studies, genetic manipulation of IL-33/ST2 axis on BALB/c background, can change susceptibility to disease in different experimental models of autoimmune diseases [20–22], and make them more "C57Bl/6 like".

The objective of this study was to compare adipose tissue and liver immunophenotype, the degree of adiposity, liver steatosis, inflammation and fibrosis in C57Bl/6 and BALB/c mice fed chow and high fat diet. We demonstrate inherent and high-fat diet induced differences in immune cell types in visceral adipose tissue and liver, systemic and liver cytokine profiles, blood glucose levels, degree of adiposity, liver steatosis, inflammation and fibrosis. C57Bl/6 and BALB/c mice differentially regulate the expression of genes related to lipid metabolism and fibrosis in liver in response to high fat feeding. Strain-dependent changes in metabolic variables and immune cell composition in metabolic tissues need to be considered in designing metabolic studies, particularly in studying fibrotic NASH.

## Materials and Methods

### Experimental mice and study design

Male C57Bl/6 mice and BALB/c mice, 8-weeks old, were purchased from Military Medical Academy, Belgrade, Serbia and accommodated in our animal facilities under standard laboratory conditions in a temperature-controlled environment with a 12 h light/darkness cycle and received water and standard diet (10% calories from fat, Mucedola, Italy) or high fat diet (HFD, 60% calories from fat, Mucedola, Italy) *ad libitum*. Animals were fed for 24 weeks. The total number of mice used in experiments was 50. The general health and pathogen status of the mice was monitored by animal farm veterinary every two weeks. During experiment, two animals were found dead, both from C57Bl/6 group on high fat diet. Mice were sacrificed by cervical dislocation and blood samples, visceral adipose tissue and liver were collected for further analyses.

### Ethics Statement

This study was carried out in strict accordance with the recommendations stated in the European Union Directive 2010/63/EU. All animal procedures were approved by the Ethical committee of the Faculty of Medical Sciences, University of Kragujevac (Permit Number: 01-2759/3). All efforts were made to minimize animal suffering.

### Metabolic parameters

Body weights and fasting blood glucose levels were measured once every 4 weeks. Mice were fasted for 4h, and glucose levels (mmol/L) were determined using the Accu-Chek Performa glucometer (Roche Diagnostics, Mannheim, Germany). Serum concentrations of triglycerides, total cholesterol, HbA1c, AST and ALT were measured using the Olympus AU600 Chemistry Immuno Analyzer (Olympus, Tokyo, Japan). Fasting insulin levels in sera was determined using the Mouse Insulin ELISA Kit (Alpco, Salem, NH, USA).

### Histological analysis of visceral adipose tissue

Visceral adipose tissue (VAT) including epididymal and adipose tissue around internal organs was excised and weighed. VAT was fixed in 10% buffered formalin, embedded in paraffin and tissue sections (5μm) stained with hematoxylin–eosin (H&E). For adipocyte size measurement fifty adjacent cells were analyzed from a total of five H&E stained sections from each mouse using light microscope (BX51; Olympus) equipped with a digital camera and ImageJ software. Data are expressed as mean adipocyte diameter (μm).

### Isolation of visceral adipose tissue stromal vascular fraction cells

Total visceral adipose tissue (VAT) was minced and digested using 1 mg/ml collagenase type II and 2% BSA (Sigma-Aldrich, St. Louis, MO) in phosphate buffered saline (PBS) as previously described [12]. Tissues were left in shaking water bath for 1h and then passed through a 40-mm nylon cell strainer (BD Biosciences, San Jose, CA) to enrich stromal vascular fraction cells (SVF cells).

### Liver histological analysis

Livers were excised, fixed in 10% buffered formalin and embedded in paraffin. Tissue sections (5μm) were stained with H&E and Picrosirius red as previously described [23]. Quantification of red-stained collagen in mouse liver sections stained with Picrosirius red was performed on 10 fields of a section at 10X magnification, as previously described [24].

Periodic Acid-Schiff staining was performed on paraffin embedded liver tissue sections using PAS Kit (Sigma-Aldrich, St. Louis, MO) according to manufacturer protocol. Quantification of PAS positive areas in liver sections was performed on 10 fields of a section at 20X magnification. Photomicrographs were analyzed quantitatively by image analysis in ImageJ software using color deconvolution as described previously [25].

Oil-Red-O staining was performed on liver tissue cryosections (5μm). Tissue sections were fixed in paraformaldehyde (10%), rinsed with 60% isopropanol and stained with freshly prepared Oil-Red-O working solution for 10 minutes. After rinsing with 60% isopropanol sections were counterstained with Mayer's haematoxylin and mounted with glycerin jelly. Quantification of red-stained lipids in mouse liver sections stained with Oil-Red-O was performed on 10 fields of a section at 100X magnification, as previously described [26].

Histological quantification of liver tissue inflammatory cell infiltration was done in blinded fashion by two independent observers. Analysis was performed on 10 fields of a section at 10X magnification. Inflammatory cell infiltrate was graded as follows: 0 = no foci; 1 = <2 foci per field; 2 = 2–4 foci per field; 3 = >4 foci per field, and the mean score was calculated [27]. Liver histological analysis was done on tissue sections images captured with a light microscope (BX51; Olympus) equipped with a digital camera.

### Immunohistochemistry

For immunohistochemical staining we used paraffin embedded sections (5μm) of mouse liver tissue. We performed heat mediated antigen retrieval in Citrate Buffer (pH = 6.0). Deparaffinized tissue-sections were incubated with primary mouse anti-α-SMA antibody (ab7817, Abcam, Cambridge, UK) and primary mouse anti-CD68 antibody (ab49777, Abcam). Staining was visualized by using Mouse Specific HRP/DAB Detection IHC Kit (ab64259, Abcam) and sections were counterstained with Mayer's hematoxylin. Sections were photomicrographed with a digital camera mounted on light microscope (Olympus BX51, Japan), digitized and analyzed. Analysis was performed on 10 fields of a section at 40X magnification. Results are presented as mean count of positive cells per field.

### Isolation of liver mononuclear cells

Mice were euthanized, the livers were removed and hepatic mononuclear cells were isolated. The livers were thoroughly dissected and passed through a 100-μm nylon cell strainer (BD Biosciences) and then suspended in complete RPMI 1640 medium containing 10% fetal calf serum (FCS). Cell suspensions were centrifuged at 507 rpm for 1 minute and the supernatants enriched for mononuclear cells were collected and centrifuged at 1500 rpm for 10 minutes as previously described [21]. Cell pellets were resuspended in complete RPMI 1640 medium.

### Flow cytometry

Liver and visceral adipose tissue mononuclear cells were stained with either combinations of fluorochrome-labeled primary Abs or isotype controls for 30 min at 4°C. For intracellular staining, cells were activated with PMA/ionomycin and processed as previously described [28].

Cells were labeled with fluorochrome-conjugated monoclonal antibodies: anti-mouse CD3 (557666 and 553067, BD Biosciences), CD3 (553067, BD Biosciences), CD11b, (12-0112-82, eBioscience, San Diego, CA), CD45 (553079, BD Biosciences), CD45 (A14792, Life Technologies, Carlsbad, CA), CXCR3 (562152, BD Biosciences), CD49b (108910, Biolegend, San Diego, CA), CD4 (557681, BD Biosciences), CD8 (553035, BD Biosciences), NK1.1 (550627, BD Biosciences), F4/80 (MF48020, Invitrogen), CD11c (MCD11C05, Invitrogen), IL-1β (17-7114-80, eBioscience, San Diego, CA), CD206 (ABTU0111121, R&D Systems, Minneapolis, MN), Ly-6C (Life Technologies, Carlsbad, CA). The cells were analyzed using a FACS Calibur (BD Biosciences) and FlowJo software (Tree Star).

### Expression of genes related to lipid metabolism and fibrosis in liver

Total RNA was extracted from a frozen mouse liver using TRIzol (Invitrogen, Carlsbad, CA) according to the manufacturer's instructions. Total RNA (2μg) was reverse-transcribed to cDNA using High Capacity cDNA Reverse Transcription Kit (Applied Biosystems, Foster City, California, USA). qRT-PCR was performed using Power SYBR MasterMix (Applied Biosystems) and miRNA specific primers for Procollagen, CD36, IL-13, TGF-β, Abca-1, LXRα, PPARγ, SREBP-1c and β-actin as a housekeeping gene (Table 1). qPCR reactions were initiated with a 10 minute incubation time at 95°C followed by 40 cycles of 95°C for 15 seconds and 60°C for 60 seconds in a Mastercycler ep realplex (Eppendorf, Hamburg, Germany). Relative expression of genes was calculated according to the formula 2^-(Ct-Ctactin),^ where C_t_ is the cycle threshold of the gene of interest and C_tactin_ is the cycle threshold value of the housekeeping gene (ß-actin) [29].

**Table 1:** Primers used for qRT-PCR analysis.

**β-actin**	5’- AGCTGCGTTTTACACCCTTT-3’	5’- AAGCCATGCCAATGTTGTCT-3’
**Procollagen**	5’- GCTCCTCTTAGGGGCCACT-3’	5’- CCACGTCTCACCATTGGGG-3’
**TGF-β**	5’-ATTCCTGGCGTTACCTTG-3’	5’-CTGTATTCCGTCTCCTTGGTT-3’
**IL-13**	5’-CCTGGCTCTTGCTTGCCTT-3’	5’-GGTCTTGTGTGATGTTGCTCA-3’
**Abca 1**	5′-CGCAGTGACCAGAAAACAATGTG-3′	5′-TATCAAGTAG GCAAGGGTGTGG-3′
**LXRα**	5`- ATCGCCTTGCTGAAGACCTCTG-3′	5′-GATGGGGTTGATGAACTCCACC-3′
**PPAR-γ**	5′-CCCAATGGTTGCTGATTACAAA-3′	5′-GAGGGAGTTAGAAGGTTCTTCATGA-3′
**SREBP-1c**	5′-GGAGCCATGGATTGCACATT-3′	5′-GGCCCGGGAAGTCACTGT-3′
**CD36**	5′-TCCAGCCAATGCCTTTGC-3′	5′-TGGAGATTACTTTTTCAGTGCAGA A-3′

### Cytokine measurements

The liver tissues were weighed and homogenized at 4°C. Liver homogenates were centrifuged at 14000xg for 10 min at 4°C. Supernatants were transferred to clean microcentrifuge tubes and stored at -20°C. Cytokine levels in mouse sera and supernatants of liver homogenates were determined using mouse Duoset ELISA kits for IL-6, IL-10, IL-13, IL-33, TGF-β, IFN-γ and TNF-α (R&D Systems, Minneapolis, MN, USA) according to manufacturer’s instructions.

### Statistical analysis

Statistical analysis was performed using SPSS 19.0. Data are presented as means ± SEM. Statistical significance was determined by Mann-Whitney U test, and, were appropriate, independent-sample Student's t-test. Statistical significance was assumed at p<0.05.

## Results

### Body weight and glucose metabolism in C57Bl/6 and BALB/c mice

**Fig 1:**
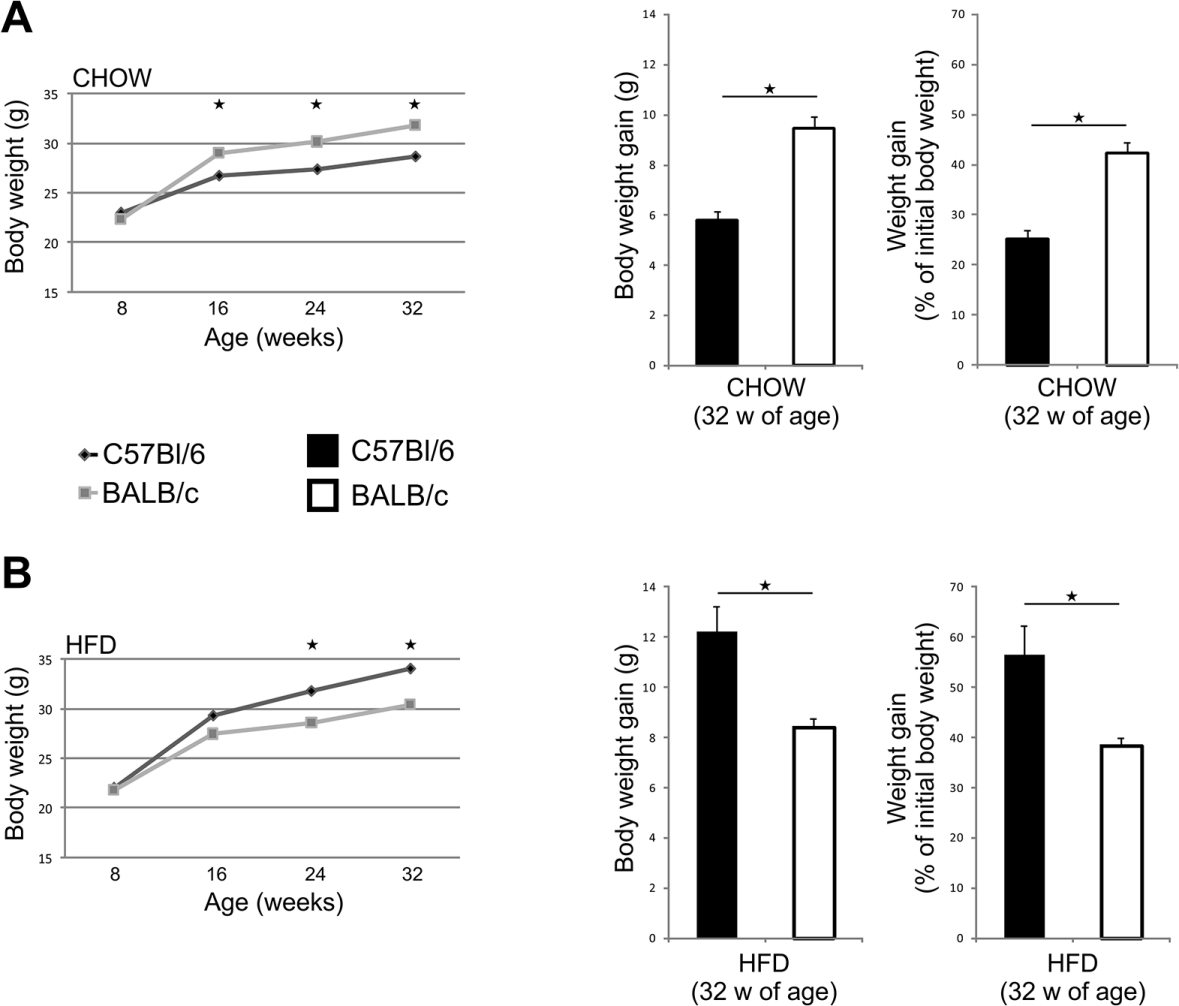
Changes in body weight of chow and HFD-fed C57Bl/6 and BALB/c mice during 32 weeks of the experiment. (A) Body weights of chow-fed C57Bl/6 mice at 16, 24 and 32 weeks of age were significantly lower than that of BALB/c mice. Body weight gain and weight gain as a percentage of initial body weight were significantly lower in chow-fed C57Bl/6 mice. (B) Body weights of HFD-fed C57Bl/6 mice at 24 and 32 weeks of age were significantly higher than that of BALB/c mice. Body weight gain and weight gain as a percentage of initial body weight were significantly higher in HFD-fed C57Bl/6 mice. The results are shown as the means ± SEM of 9–10 animals per group. *p<0.05. The results are representative of two experiments.

At the beginning of the experiment 8 weeks old C57Bl/6 and BALB/c mice had similar body weights. At 32 weeks of age, C57Bl/6 mice fed chow for 24 weeks, had significantly lower body weight (p = 0.029), body weight gain (p = 0.009) and weight gain as percentage of initial body weight (p = 0.009) in comparison with BALB/c mice (Fig 1A). However, C57Bl/6 mice fed HFD for 24 weeks had significantly higher body weight (p = 0.039), body weight gain (p = 0.009) and weight gain as percentage of initial body weight (p = 0.009) compared with HFD-fed BALB/c mice (Fig 1B).

**Fig 2:**
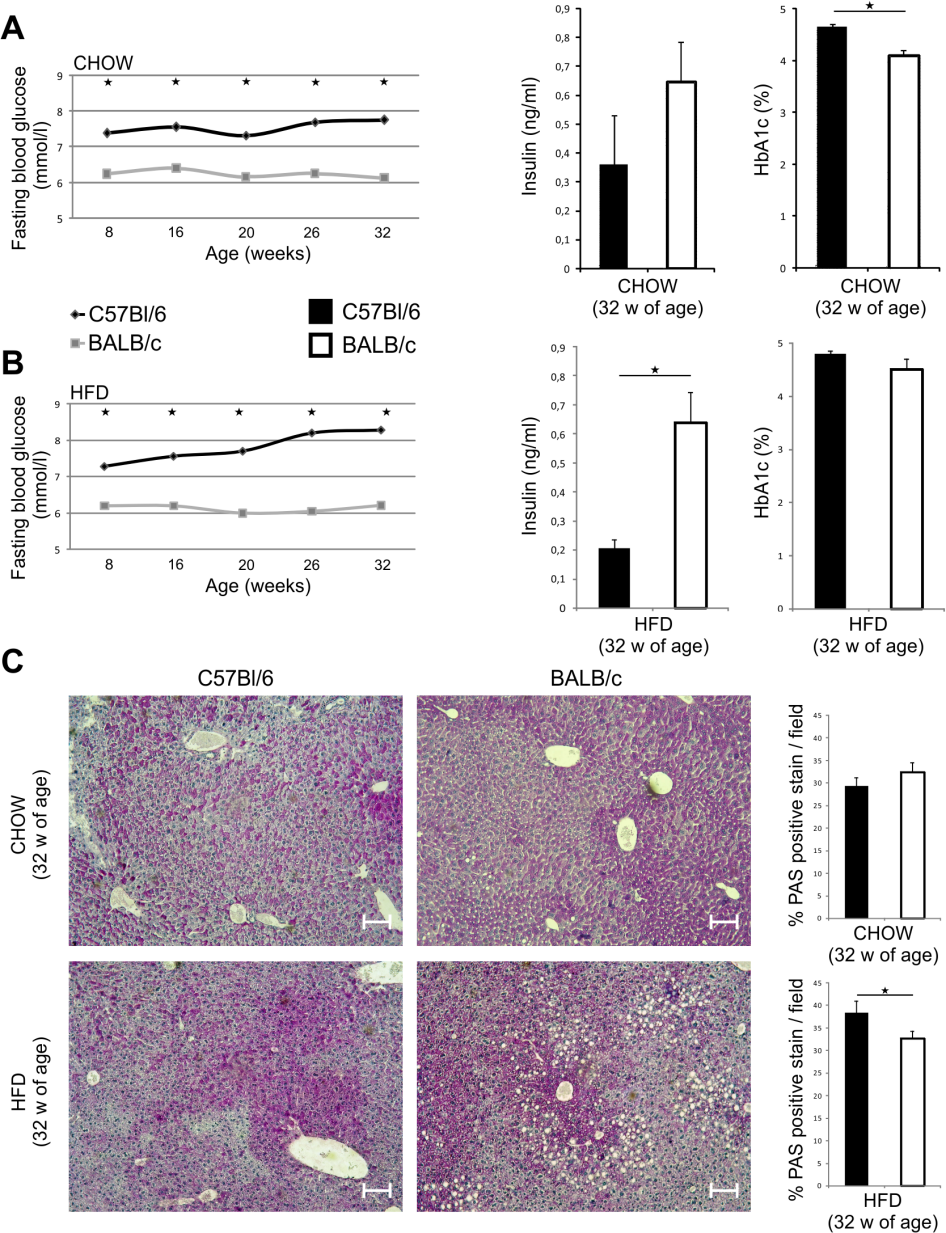
Blood glucose levels and liver glycogen storage in chow and HFD-fed C57Bl/6 and BALB/c mice. (A) Fasting blood glucose levels were significantly higher in chow-fed C57Bl/6 mice compared with chow-fed BALB/c mice. Serum insulin levels did not differ, while HbA1c was significantly higher in chow-fed C57Bl/6 mice compared with chow-fed BALB/c mice at 32 weeks of age. (B) Fasting blood glucose levels were significantly higher in HFD-fed C57Bl/6 mice compared to HFD-fed BALB/c mice. Serum insulin levels were significantly higher in HFD-fed C57Bl/6 mice compared to HFD-fed BALB/c mice, with no difference in HbA1c at 32 weeks of age. (C) Representative images of PAS staining on paraffin-embedded liver tissue sections (original magnification x10, scale bar = 100μm). Glycogen stores were significantly higher in liver of HFD-fed C57Bl/6 mice compared to HFD-fed BALB/c mice at 32 weeks of age. The results are shown as the means ± SEM for 9–10 animals per group. *p<0.05. The results are representative of two experiments.

Fasting blood glucose levels were significantly higher in C57Bl/6 mice at 8 weeks of age (p = 0.009) and at 32 weeks of age (p = 0.003) compared to chow-fed BALB/c mice. Glycosylated hemoglobin (HbA1c) was higher in chow-fed C57Bl/6 mice at 32 weeks of age (p = 0.021) compared to chow-fed BALB/c (Fig 2A). Fasting blood glucose levels were also higher in C57Bl/6 mice on HFD at 32 weeks of age compared to diet-matched BALB/c mice (p = 0.009), while fasting serum insulin level was lower (p = 0.006) (Fig 2B). In comparison with BALB/c mice, C57Bl/6 mice had higher liver glycogen content at 8 weeks of age (p = 0.007) (data not shown) and at 32 weeks of age on HFD regimen (p = 0.013) (Fig 2C).

### Visceral fat mass and adipose tissue associated immune cells in C57Bl/6 and BALB/c mice

**Fig 3:**
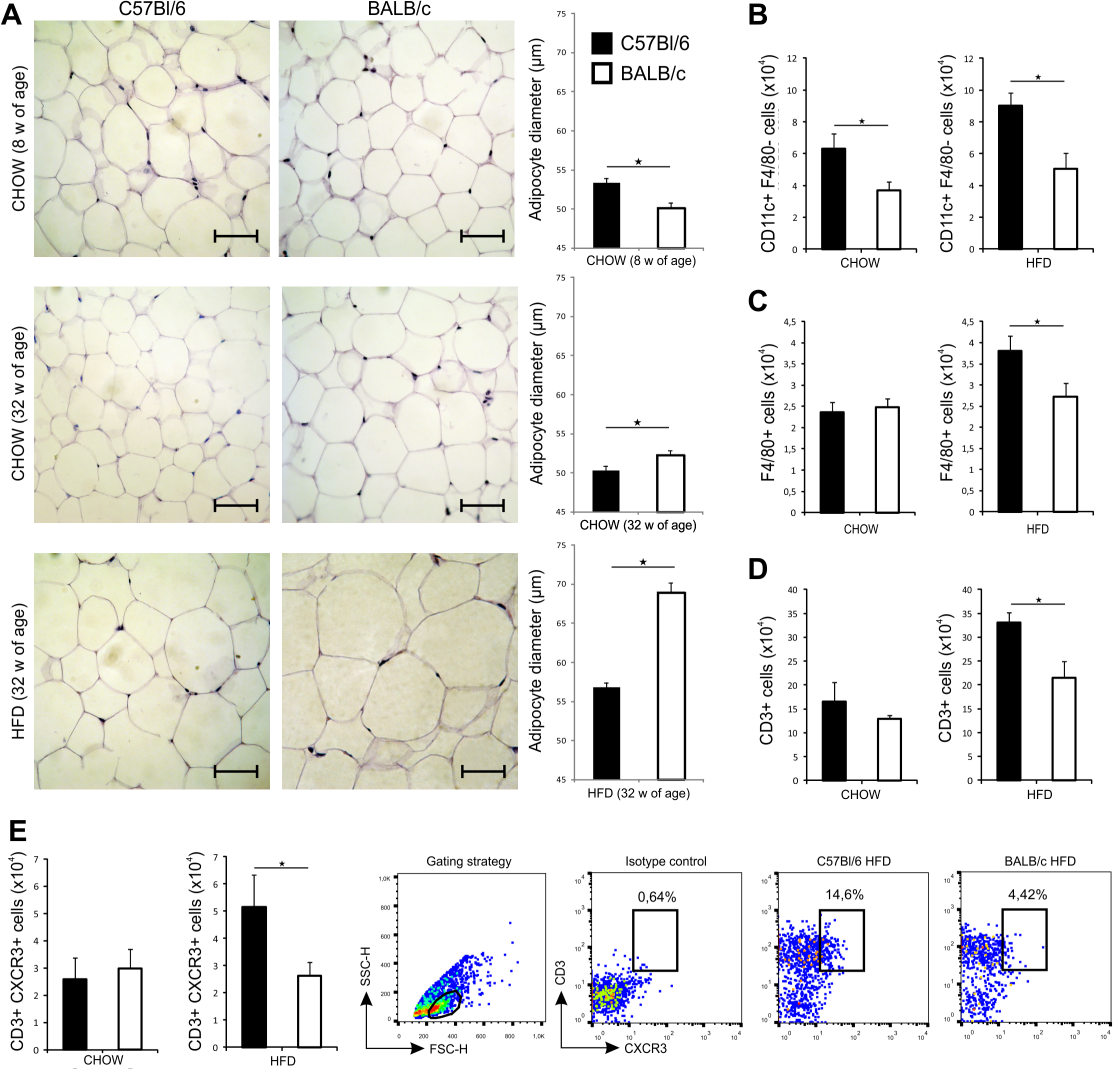
Adipocyte size and phenotypic analysis of immune cells in VAT in chow and HFD-fed C57Bl/6 and BALB/c mice. (A) Representative images of hematoxylin-eosin staining (original magnification x40, scale bar = 50μm) of paraffin-embedded visceral adipose tissue sections. Mean adipocyte diameter was significantly higher in 8-weeks old chow-fed C57Bl/6 mice, while significantly lower in 32 weeks old chow and HFD-fed mice, compared with diet-matched BALB/c mice. (B) Number of CD11c^+^F4/80^-^ DCs in VAT is significantly higher in chow- and HFD-fed C57Bl/6 mice compared with diet-matched BALB/c mice at 32 weeks of age. (C) Number of F4/80^+^ macrophages is significantly higher in HFD-fed C57Bl/6 mice compared to HFD-fed BALB/c mice at 32 weeks of age. (D) Number of VAT associated CD3^+^ lymphocytes is significantly higher in HFD-fed C57Bl/6 mice compared to HFD-fed BALB/c mice at 32 weeks of age. (E) Number of VAT associated CD3^+^CXCR3^+^ lymphocytes is significantly higher in HFD-fed C57Bl/6 mice compared to HFD-fed BALB/c mice at 32 weeks of age. Gating strategy and representative FACS plots of CD3^+^CXCR3^+^ cells in VAT of C57Bl/6 and BALB/c mice on HFD. The results are shown as the means ± SEM of 9–10 animals per group. *p<0.05. The results are representative of two experiments.

The amount of visceral adipose tissue was significantly higher in C57Bl/6 mice at 8 weeks (p = 0.003) and at 32 weeks of age on chow (p = 0.009) and HFD (p = 0.008) compared to diet-matched BALB/c mice (Table 2). At 32 weeks of age body visceral fat percentage was significantly higher in C57Bl/6 mice after 24 weeks on standard diet (p = 0.05) and HFD (p = 0.05) (Table 2). Serum triglycerides (p = 0.029) and total cholesterol (p = 0.012) levels were significantly lower in C57Bl/6 mice compared with BALB/c mice (Table 2). The mean size of adipocytes was significantly higher in C57Bl/6 mice than in BALB/c mice at 8 weeks of age (p = 0.020), but significantly lower at 32 weeks of age on chow (p = 0.001) and HFD (p = 0.001) (Fig 3A).

**Table 2:** Amount of VAT and lipid profile in C57Bl/6 and BALB/c mice.

	8 weeks of age CHOW		32 weeks of age CHOW		32 weeks of age HFD	
	C57Bl/6	BALB/c	C57Bl/6	BALB/c	C57Bl/6	BALB/c
Visceral adipose tissue (g)	0,23 ± 0,03	0,17 ± 0,06*	0,46 ± 0,02	0,34 ± 0,04*	1,74 ± 0,24	0,70 ± 0,08*
Visceral fat mass (% body weight)	0,98 ± 0,13	0,70 ± 0,23	1,53 ± 0,05	1,16 ± 0,14*	5,09 ± 0,62	2,25 ± 0,24*
Total cholesterol (mmol/l)	3,07 ± 0,29	4,73 ± 0,40*	3,14 ± 0,25	3,93 ± 0,32*	4,76 ± 0,13	6,02 ± 0,27*
Triglycerides (mmol/l)	0,92 ± 0,02	1,17 ± 0,19*	1,68 ± 0,14	1,85 ± 0,15*	1,34 ± 0,12	1,94 ± 0,06*

The amount of visceral adipose tissue was significantly higher in C57Bl/6 mice at 8 weeks of age, and in chow and HFD-fed mice at 32 weeks of age. Visceral fat mass (percentage of total body weight) was significantly higher in C57/Bl/6 mice at 32 weeks of age on chow and HFD. Total cholesterol levels and triglyceride levels were significantly lower in C57Bl/6 mice at 8 weeks of age and at 32 weeks of age on chow and HFD. The results are shown as the means ± SEM for 9–10 animals per group.

*p<0.05. The results are representative of two experiments.

We next analyzed nonlymphoid accessory cells in visceral adipose tissue, the cells that affect polarization and function of T cells. The number of VAT associated CD11c^+^ dendritic cells (DCs) was significantly higher in C57Bl/6 fed chow or HFD compared to diet-matched BALB/c mice (Fig 3B). At 32 weeks of age, the number of F4/80^+^ macrophages in VAT was significantly higher in C57Bl/6 mice fed HFD compared to diet-matched BALB/c mice, while there were no difference in mice fed chow at the same time point (Fig 3C).

Further, C57Bl/6 mice fed HFD had higher number of CD3^+^ T cells and CD3^+^CXCR3^+^ T cells compared to HFD-fed BALB/c mice (Fig 3D and 3E), while there were no differences in the frequencies of these cell populations in mice fed chow at 32 weeks of age. HFD led to an increase of the number of NKT and NK cells in VAT in both mouse strains with no significant differences between C57Bl/6 and BALB/c mice (data not shown).

### Immune cell composition in liver of C57Bl/6 and BALB/c mice

**Fig 4:**
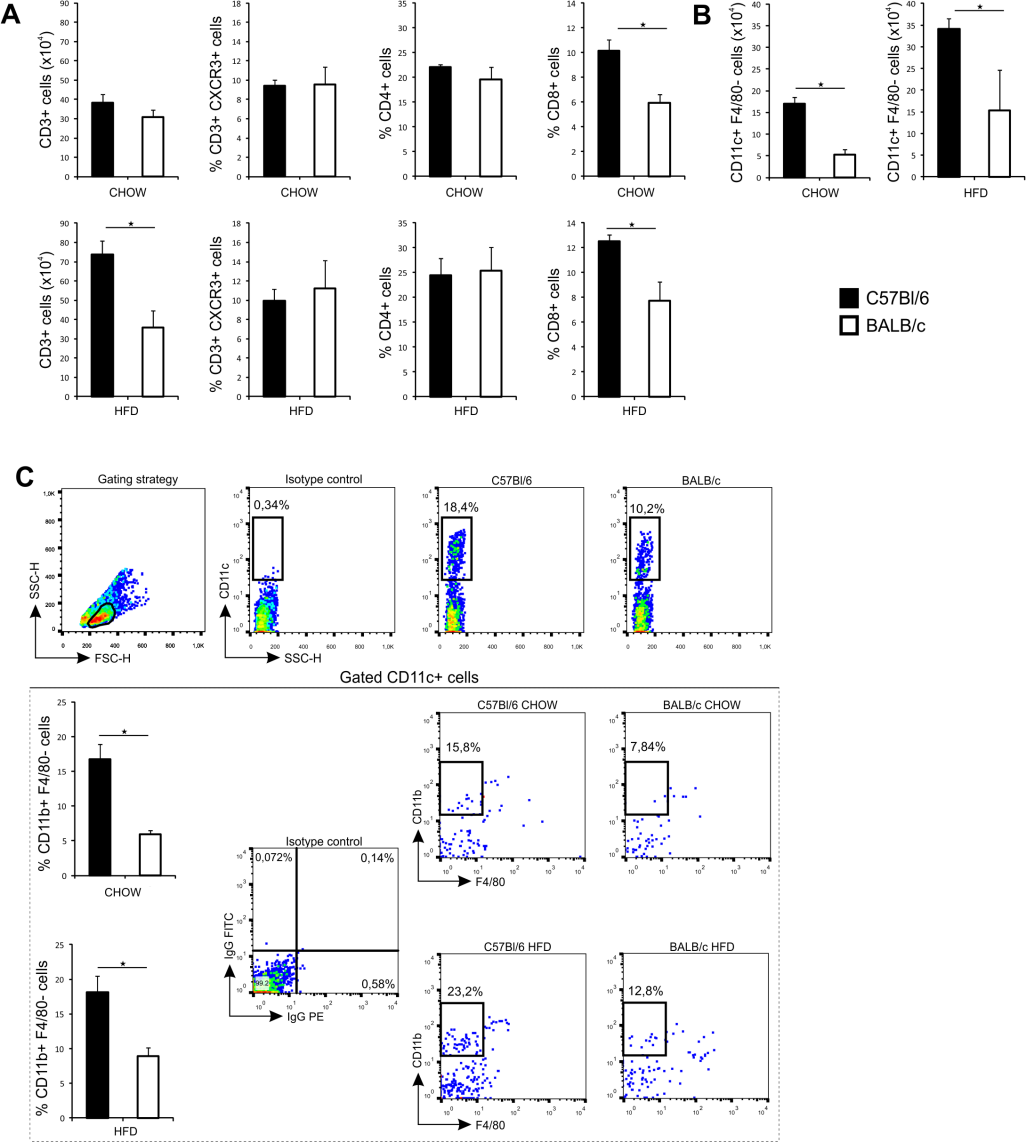
Phenotypic analysis of immune cells in liver in chow and HFD-fed C57Bl/6 and BALB/c mice. (A) Number of CD3^+^ cells in liver was significantly higher in HFD-fed C57Bl/6 mice. Percentage of CD8^+^ cells was significantly higher in chow- and HFD-fed C57Bl/6 mice compared with diet-matched BALB/c mice at 32 weeks of age. (B) Number of CD11c^+^ F4/80^-^ cells in liver was significantly higher in chow- and HFD-fed C57Bl/6 mice compared with diet-matched BALB/c mice at 32 weeks of age. (C) Among gated CD11c^+^ cells, the percentage of CD11b^+^F4/80^-^ DCs was significantly higher in liver of C57Bl/6 mice on both diets. Gating strategy, isotype controls and representative FACS plots of gated CD11c^+^CD11b^+^F4/80^-^ cells in liver of C57Bl/6 and BALB/c mice on both diets. The results are shown as the means ± SEM of 9–10 animals per group. *p<0.05. The results are representative of two experiments.

Livers of C57Bl/6 and BALB/c mice fed chow contained similar number of CD3^+^ lymphocytes and percentages of CD4^+^ cells and CD3^+^CXCR3^+^ T cells. Livers of C57Bl/6 mice fed HFD contained higher number of CD3^+^ cells. The higher percentage of CD8^+^ T cells were found in the liver of C57Bl/6 mice fed chow or HFD compared to diet-matched BALB/c mice (Fig 4A).

**Fig 5:**
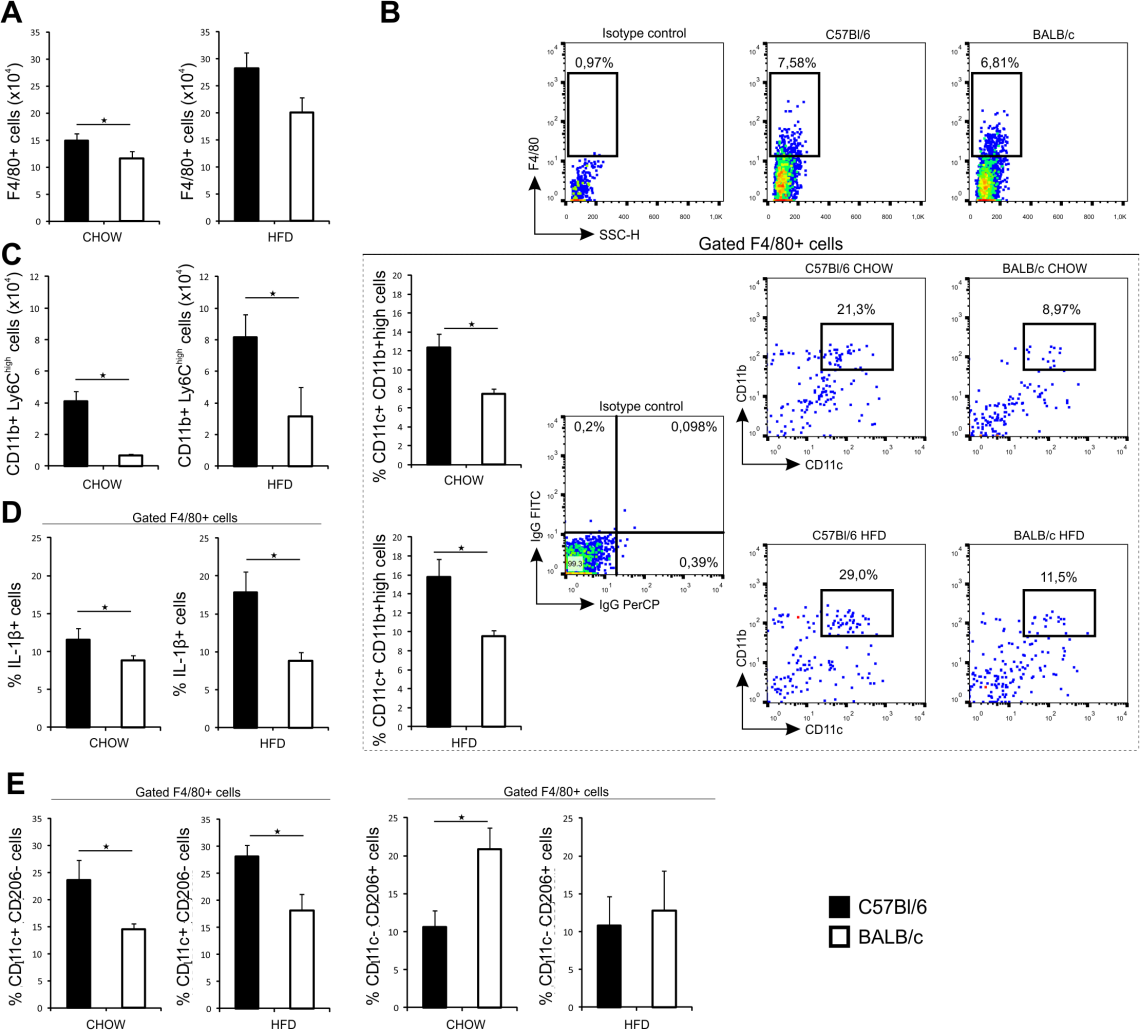
Phenotypic analysis of macrophages in liver in chow and HFD-fed C57Bl/6 and BALB/c mice. (A) Number of F4/80^+^ macrophages was significantly higher in chow-fed C57Bl/6 mice compared to chow-fed BALB/c mice at 32 weeks of age. (B) Percentage of triple positive F4/80^+^ CD11b^high^ CD11c^+^ macrophages was significantly higher in liver of chow- and HFD-fed C57Bl/6 mice compared with diet-matched BALB/c mice at 32 weeks of age. Gating strategy, isotype controls and representative FACS plots of triple positive F4/80^+^ CD11b^high^ CD11c^+^ cells in liver of C57Bl/6 and BALB/c mice on both diets. (C) Number of CD11b^+^Ly6C^high^ cells was significantly higher in liver of chow- and HFD-fed C57Bl/6 mice compared with diet-matched BALB/c mice at 32 weeks of age. (D) Percentage of IL-1β expressing F4/80^+^ macrophages was significantly higher in liver in chow- and HFD-fed C57Bl/6 mice compared with diet-matched BALB/c mice at 32 weeks of age. (E) Percentage of CD11c^+^CD206^-^ cells among gated F4/80^+^ cells was significantly higher in liver in chow- and HFD-fed C57Bl/6 mice compared with diet-matched BALB/c mice at 32 weeks of age. Percentage of CD11c^-^CD206^+^ cells among gated F4/80^+^ cells was significantly lower in liver of chow-fed C57Bl/6 mice. The results are shown as the means ± SEM of 9–10 animals per group. *p<0.05. The results are representative of two experiments.

The number of CD11c^+^ DCs and the percentage of myeloid DCs were significantly higher in C57Bl/6 mice fed chow or HFD compared to diet-matched BALB/c mice at 32 weeks of age (Fig 4B and 4C). The number of F4/80^+^ and the percentage of proinflammatory F4/80^+^CD11b^high^CD11c^+^ macrophages were higher in C57Bl/6 mice on both diets as shown in Fig 5A and 5B. Similarly, liver proinflammatory CD11b^+^Ly6C^high^ monocytes and IL-1β expressing F4/80^+^ macrophages were higher in C57Bl/6 mice on both diets compared with diet-matched BALB/c mice (Fig 5C and 5D). The percentage of M1 macrophages (F4/80^+^CD11c^+^CD206^-^) was significantly higher in liver of C57Bl/6 mice compared to BALB/c mice. In mice fed chow, the percentage of M1 macrophages (F4/80^+^CD11c^+^CD206^-^) was significantly higher in liver of C57Bl/6 mice, while the percentage of M2 (F4/80^+^CD11c^-^CD206^+^) macrophages were significantly higher in liver of BALB/c mice. In HFD-fed mice, C57Bl/6 mice had higher proportion of M1 macrophages in liver with no difference in M2 macrophages compared to BALB/c mice (Fig 5E).

HFD increased the numbers of F4/80^+^CD11b^-^ Kupffer cells in both mouse strains with no difference between C57Bl/6 and BALB/c mice (data not shown).

### Liver steatosis, inflammation and fibrosis in C57Bl/6 and BALB/c mice

**Fig 6:**
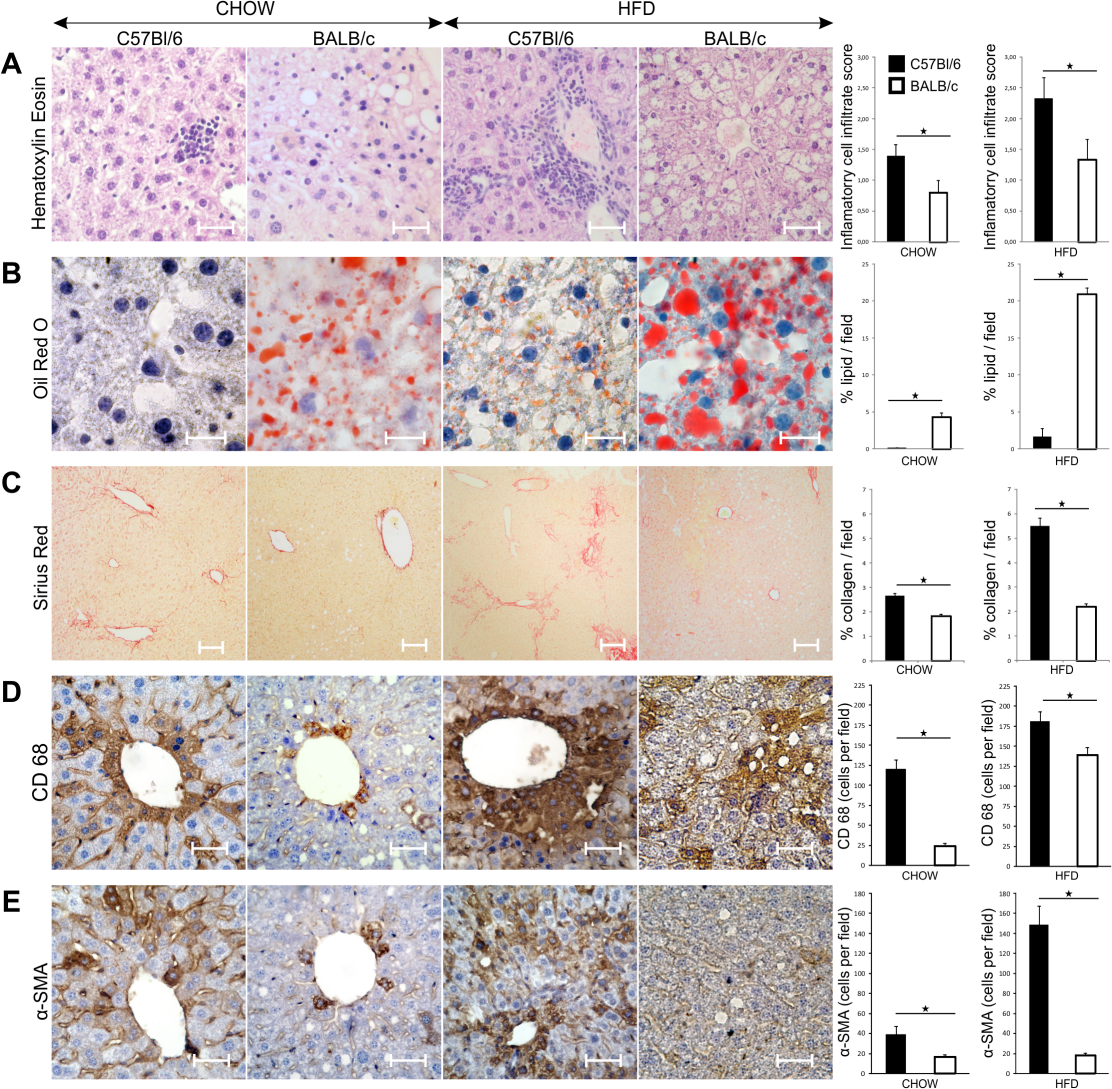
Liver inflammation, steatosis and fibrosis in chow and HFD-fed C57Bl/6 and BALB/c mice. (A) Representative images of HE staining of paraffin-embedded liver tissue sections (original magnification x40, scale bar = 50μm). Results are presented as inflammatory infiltrate score according to NASH score system. Inflammatory infiltrate score was significantly higher in chow- and HFD-fed C57Bl/6 mice compared with diet-matched BALB/c mice at 32 weeks of age. (B) Representative images of Oil Red O staining on frozen liver sections (original magnification x100, scale bar = 25μm). Results are presented as percentage of red stained area relative to the total section area. Percentage of stained lipids was significantly lower in chow- and HFD-fed C57Bl/6 mice compared with diet-matched BALB/c mice at 32 weeks of age. (C) Representative images of Sirius Red staining on paraffin-embedded liver tissue sections (original magnification x10, scale bar = 100μm). Results are presented as percentage of red stained area relative to the total section area. Percentage of stained collagen fibers was significantly higher in chow- and HFD-fed C57Bl/6 mice compared with diet-matched BALB/c mice at 32 weeks of age. (D) Representative images of CD68 IHC staining on paraffin-embedded liver sections (original magnification x40, scale bar = 50μm). Number of CD68 positive cells per field was significantly higher in chow- and HFD-fed C57Bl/6 mice compared with diet-matched BALB/c mice at 32 weeks of age. (E) Representative images of αSMA IHC staining on paraffin-embedded liver sections (original magnification x40, scale bar = 50μm). Number of α-SMA positive cells per field was significantly higher in chow- and HFD-fed C57Bl/6 mice compared with diet-matched BALB/c mice at 32 weeks of age. The results are shown as the means ± SEM of 9–10 animals per group. *p<0.05. The results are representative of two experiments.

Liver inflammation, as evaluated by inflammatory cell infiltrate score, was significantly more pronounced in livers of C57Bl/6 mice compared with BALB/c mice fed chow (p = 0.004) and HFD (p = 0.000) at 32 weeks of age (Fig 6A). C57Bl/6 mice had no signs of liver steatosis, while in BALB/c mice intralobular micro and macrosvesicular steatosis was present and visible in hematoxylin-stained sections. Semiquantitative analysis of accumulated lipids in liver tissue sections stained with Oil-Red-O demonstrated statistically significantly lower liver steatosis in C57Bl/6 mice compared to BALB/c mice at 8 weeks (p = 0.02) (data not shown) and 32 weeks of age on chow (p = 0.000) and HFD (p = 0.000) (Fig 6B). The degree of liver fibrosis, quantified on the basis of Picrosirius red staining of collagen, was significantly higher in C57Bl/6 mice than in the BALB/c mice at 8 weeks (p = 0.000) (data not shown) and at 32 weeks of age on chow (p = 0.000) and HFD (p = 0.000) (Fig 6C). Liver fibrosis in HFD fed C57Bl/6 mice was diffusely distributed in the portal tracts and in perisinusoidal and centrolobular regions, while rare signs of collagen deposition were mainly located in the portal tracts and pericellularly in diet-matched BALB/c mice.

The number of CD68^+^ macrophages (Fig 6D) was significantly higher in liver of C57Bl/6 mice at 32 weeks of age on standard and HFD. The number of α-SMA positive liver myofibroblasts was significantly higher in C57Bl/6 mice compared to BALB/c mice fed chow (p = 0.000) and HFD (p = 0.000) at 32 weeks of age (Fig 6E).

Markers of liver injury, serum aspartate transaminase (AST) and alanine transaminase (ALT) levels were not significantly different between the mice strains fed chow. At 32 weeks of age, serum AST and ALT levels were significantly higher in C57Bl/6 mice (p = 0.013 and p = 0.040, respectively) compared to BALB/c mice, both on HFD (data not shown).

### Expression of genes related to liver lipid metabolism and fibrosis and cytokine profiles in C57Bl/6 and BALB/c mice

**Fig 7:**
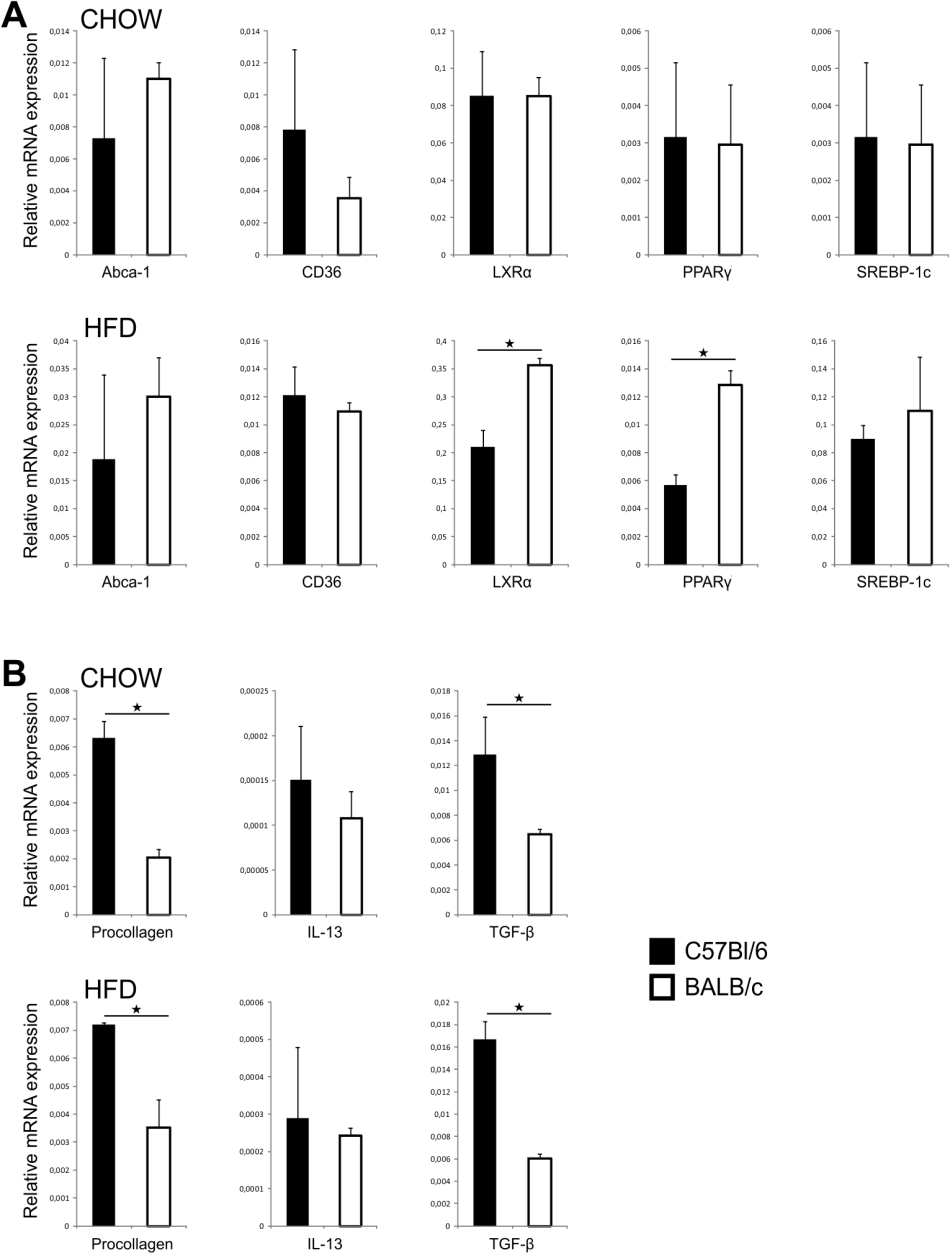
Liver gene expression in chow and HFD-fed C57Bl/6 and BALB/c mice. (A) Expression of genes related to lipid metabolism in liver in chow and HFD-fed C57Bl/6 and BALB/c mice. Expression of LXRα and PPARγ was significantly lower in C57Bl/6 mice mice on HFD. (B) Expression of profibrogenic genes in liver in chow and HFD-fed C57Bl/6 and BALB/c mice. Expression of procollagen and TGF-β was significantly higher in C57Bl/6 mice on both diets. The results are shown as the means ± SEM of 9–10 animals per group. *p<0.05. The results are representative of two experiments.

There were no significant differences in genes related to lipid metabolism in livers of C57Bl/6 and BALB/c mice fed chow. Significantly increased expression of genes for oxysterol receptor LXR-alpha (LXR-α) and peroxisome proliferator-activated receptor gamma (PPAR-γ) was observed in BALB/c mice on HFD at 32 weeks of age on HFD compared to diet-matched C57Bl/6 mice, while there were no differences in mRNA expression for ATP binding cassette sub-family A member 1 (Abca-1), sterol regulatory element-binding protein 1 (SREBP-1c) and CD36 (Fig 7A).

The expression of liver fibrosis related collagen alpha 1 chain precursor (procollagen) and TGF-β precursor genes were significantly increased in liver of C57Bl/6 mice fed chow and HFD compared to diet-matched BALB/c mice (all p<0.05) (Fig 7B).

**Fig 8:**
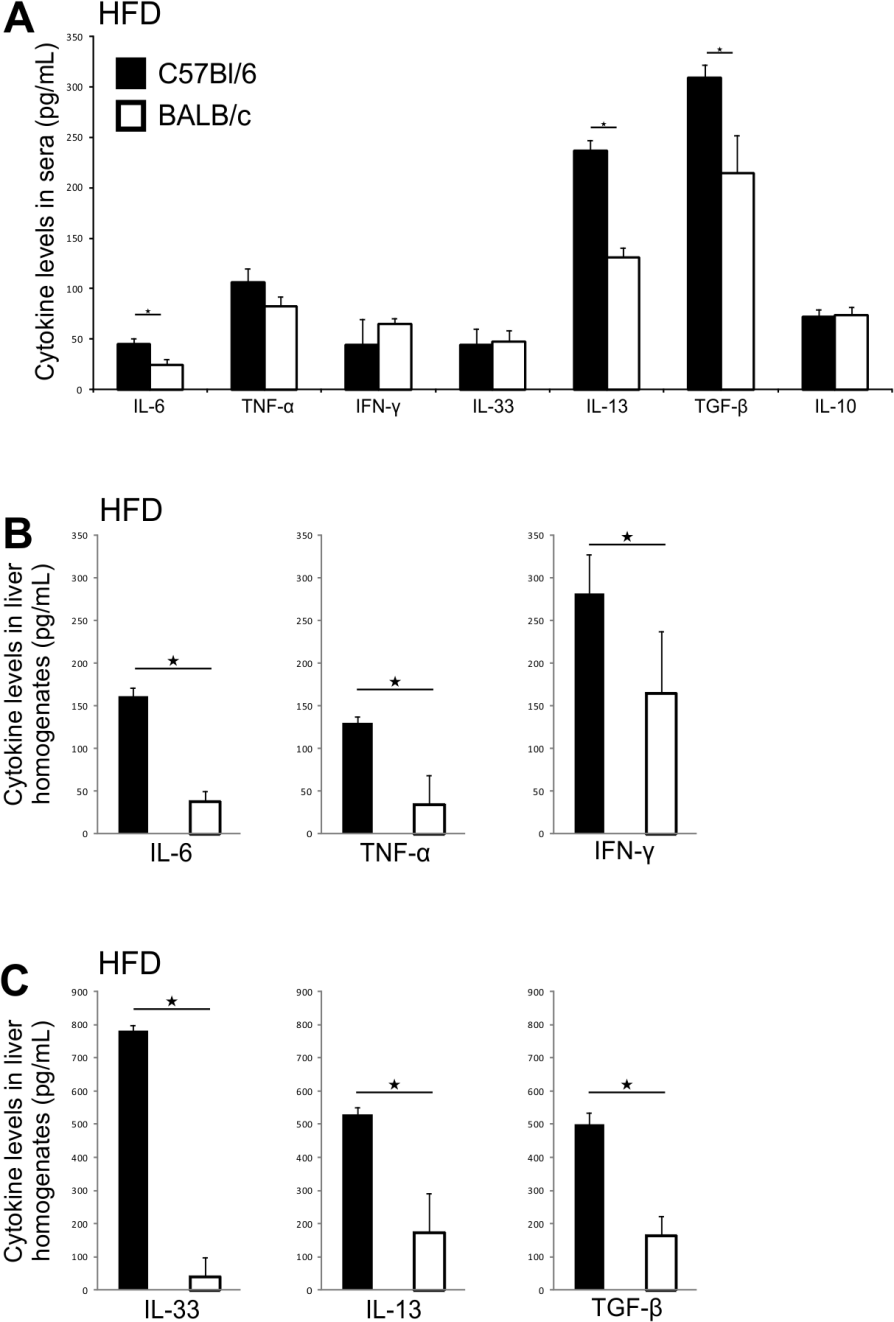
Cytokine profiles in chow and HFD-fed C57Bl/6 and BALB/c mice. (A) Serum cytokines levels in HFD-fed C57Bl/6 and BALB/c mice. IL-6, IL-13 and TGF-β were significantly higher in C57Bl/6 mice on HFD. (B) Proinflammatory cytokines levels in liver homogenates in HFD-fed C57Bl/6 and BALB/c mice. IL-6, TNF-α and IFN-γ were significantly higher in C57Bl/6 mice on HFD. (C) Profibrogenic cytokines levels in liver homogenates of HFD-fed C57Bl/6 and BALB/c mice. IL-33, IL-13 and TGF-β were significantly higher in C57Bl/6 mice on HFD. The results are shown as the means ± SEM for 9–10 animals per group. *p<0.05. The results are representative of two experiments.

We next analyzed proinflammatory and profibrotic cytokine profiles in mouse sera and supernatants of liver homogenates. The levels of IL-6, IL-13 and TGF-β were significantly higher in the sera of C57Bl/6 mice than in BALB/c mice at 32 weeks of age on chow (data not shown) and HFD (Fig 8A). In HFD-fed mice higher levels of proinflammatory IL-6, TNF-α and IFN-γ, and profibrogenic IL-33, IL-13 and TGF-β were found in liver homogenates of C57Bl/6 mice compared to BALB/c mice (Fig 8B and 8C).

## Discussion

Th1-type immune response predominates in various inflammatory diseases. In this study, we demonstrate that C57Bl/6 mouse strain which is prone to inflammation in various experimental models of autoimmune diseases, also have pronounced high-fat diet induced liver inflammation. Here, specifically in comparison to Th2-dominant BALB/c mice, we show inflammatory and fibrotic pathology in liver of C57Bl/6 mice in response to obesogenic HFD. In contrast, BALB/c mice developed less inflammatory response and fibrosis in liver, but exhibited enhanced hepatic steatosis.

C57Bl/6 and BALB/c differ in Th1 and Th2-type immune responses. In C57Bl/6 mice Th1 immune response and IFNγ production is dominant, while BALB/c strain easily mount Th2 immune response [30]. The link between immune correlates and metabolic variables in these mouse strains has not been defined. Therefore, we aimed to dissect constitutive differences in metabolic variables in C57Bl/6 and BALB/c mice on standard diet and in response to long term high fat feeding, and to explore their association with adipose tissue and liver immune cell composition.

Both mouse strains gained body weight and visceral fat at 32 weeks of age on chow and HFD. BALB/c mice fed chow had significantly higher body weight and body weight gain, but lower amount of visceral adipose tissue than C57Bl/6 mice. C57Bl/6 mice were prone to weight gain on HFD, and had higher amount of visceral adipose tissue than BALB/c mice (Fig 1 and Table 2). C57Bl/6 mice had higher blood glucose levels, HbA1c and liver glycogen stores compared with BALB/c strain. We show that adipose tissue of C57Bl/6 mice contained higher number of CD3^+^ lymphocytes and CD3^+^CXCR3^+^ T cells (Fig 3D and 3E). Chemokine receptor CXCR3 is expressed primarily on activated T cells, preferentially Th1 and NKT cells [31–32]. Our data might suggest increased number of Th1-type cells in VAT, which is consistent with previous studies that demonstrated a skew of T cells toward a Th1 phenotype in obese adipose tissue [33–36]. In support to enhanced inflammation in adipose tissue of C57Bl/6 mice on HFD, are the data of increased number of CD11c^+^ DCs and F4/80^+^ macrophages, which was not observed in VAT of BALB/c mice (Fig 3B and 3C). The mean size of adipocytes was significantly higher in BALB/c mice compared to C57Bl/6 mice on chow and HFD (Fig 3A), in which a greater number of adipocytes with different sizes were present. This finding might indicate the process of ongoing HFD-induced adipogenesis and adipocyte hyperplasia in C57Bl/6 mice compared to BALB/c mice. Growth of adipose tissue occurs by adipocytes hyperplasia and hypertrophy. Adipocyte hypertrophy often precedes adipocyte hyperplasia which is induced by the release of autocrine and paracrine growth factors in adipose tissue [37–39]. We assume that expansion of adipose tissue in BALB/c mice is achieved primarily by an increase in adipocyte size, while in C57BL/6 mice by an increase in adipocyte number.

We show inherent differences in immune cell types in livers which regulate inflammation, cytokine profiles and fibrosis in C57Bl/6 and BALB/c mice (Fig 4 and Fig 5). Livers of C57Bl/6 mice contained higher proportions of myeloid DCs and proinflammatory macrophages and monocytes and higher levels of proinflammatory cytokines than BALB/c mice.

We demonstrate that liver steatosis is more pronounced in BALB/c mice compared to C57Bl/6 mice which had scarce hepatic lipid accumulation on both diets (Fig 6B). Our finding of marked lipid accumulation in liver of BALB/c mice is in accordance with previous studies that demonstrated particularly pronounced HFD-induced liver steatosis in this mouse strain [40]. In contrast to our and other studies [41, 42], Montgomery et al. [43] reported that BALB/c mice failed to accumulate lipids in liver which might explain their ability to maintain glucose tolerance and insulin action when fed HFD. In our study BALB/c mice had higher serum levels of triglycerides and total cholesterol which is in agreement with reported studies [44, 45]. However, it should be noted that Montgomery et al. used HFD containing 45% of calories from fat for 8 weeks which is significantly shorter period of time in comparison to 24 weeks of high fat feeding in our study. Similarly to our study, HFD-induced weight gain was not significant in BALB/c mice while it was significantly increased in C57Bl/6 mice which also exhibited the increase of epididimal fat, glycemia and increased F4/80 and CD11c mRNA expression in adipose tissue [43].

Several genes involved in lipogenesis were significantly up regulated in BALB/c mice in response to high fat feeding in our study (Fig 7A). Liver X receptor alpha (LXRα) and peroxisome proliferator-activated receptor gamma (PPARγ) are involved in the control of cholesterol and lipid metabolism in liver and their upregulation is associated with prominent steatosis that we demonstrated in BALB/c mice [46, 47]. Interestingly no difference in the expression of genes related to lipid metabolism in liver of chow-fed BALB/c mice could be observed despite the presence of prominent steatosis.

Although enhanced inflammation in liver of C57Bl/6 mice and proinflammatory cytokines such as IL-1β may affect lipid metabolism and increase fatty acid biosynthesis in liver [48], we showed that steatosis in C57Bl/6 mice was scarce. At present, it is not clear which metabolic pathways is responsible for the observed differences in the hepatic accumulation of lipids in C57Bl/6 and BALB/c mice.

Liver steatosis in BALB/c mice might represent the “first hit “in the development of NASH [49]. Thus, it could be speculated that “second hit", i.e. inflammatory mediators required for the development of fibrosis are attenuated in BALB/c mice most likely due to the prevalence of Th2/Treg cells in liver [50–52]. Indeed, the selective histochemical staining of collagen fibers has identified a significantly more pronounced fibrosis in C57Bl/6 mice, while BALB/c mice had no signs of liver collagen deposition (Fig 6C). In C57Bl/6 mice liver fibrosis was diffuse and localized in the portal tracts, perisinusoidaly and centrolobularly. In comparison to BALB/c mice livers of C57Bl/6 mice contained higher proportions of proinflammatory CD11c^+^CD11b^high^ myeloid DCs, CD11b^+^Ly6C^high^ monocytes and IL-1β producing F4/80^+^ macrophages, which frequencies were markedly increased in response to high fat feeding. Although classification of liver macrophages into M1 and M2 subpopulation does not reflect their complex role in liver pathology, we show that C57Bl/6 mice on both diets have higher number of hepatic F4/80^+^CD11c^+^CD206^-^ M1 macrophages, which are closely linked to polarization towards Th1-type immune response. In contrast, BALB/c mice fed chow had more numerous F4/80^+^CD11c^-^CD206^+^ M2 macrophages. Increased numbers of CD68^+^ cells and alpha-smooth muscle actin (α-SMA) positive cells were present in livers of C57Bl/6 mice vs. BALB/c mice and their number further increased in Th1 dominant strain in response to high fat feeding. CD68 is a marker of macrophage lineage cells and is expressed in activated Kupffer cells in liver fibrosis [53–55]. Expression of α-SMA is a reliable marker of activated hepatic stellate cells which mediate fibrous tissue formation [56]. Moreover, livers of C57Bl/6 mice contained higher levels of proinflammatory/profibrogenic cytokines and upregulated profibrogenic genes in comparison with BALB/c mice (Fig 7 and Fig 8). Namely, in contrast to BALB/c mice, liver tissue homogenates in C57Bl/6 mice fed HFD had increased profibrogenic cytokines IL-33, TGF-β and IL-13 and higher procollagen mRNA expression.

We present data that show constitutive differences in immunometabolic phenotype of two mouse strains that were emphasized on high fat diet regime. Increased adiposity in C57Bl/6 mice was not accompanied with liver steatosis and upregulation of genes related to lipid metabolism in liver, but with increased liver inflammation and fibrosis. In contrast to C57Bl/6 mice, BALB/c mice exhibited prominent liver steatosis accompanied with markedly increased expression of some of the lipid metabolism genes, but the liver inflammation and fibrosis were attenuated. Thus, we provide evidence of differential regulation of visceral adiposity, liver steatosis, inflammation and fibrosis in Th1 and Th2 dominant mouse strains which could be related to differences in immunoinflammatory response to high fat feeding in C57Bl/6 and BALB/c mice.

## Conclusion

We show dissociation of liver steatosis and fibrosis related to Th1 and Th2 dominance in mice. The obtained data show differences in immune cell types in visceral adipose tissue and liver, proinflammatory and profibrotic cytokine levels and expression of hepatic genes related to lipid metabolism and fibrogenesis in C57Bl/6 and BALB/c mice.

In summing up, we show inherent differences in metabolic variables, liver steatosis, inflammation and fibrosis between C57Bl/6 and BALB/c mice, prototypical Th1 and Th2 mouse strains on standard nutrition. These strain-dependent immunometabolic differences between two mouse strains were emphasised in response to obesogenic high fat diet feeding. Inherent metabolic and immune response characteristics across different mouse strains due to their genetic background need to be considered in studies of metabolic disorders.
